# Detection of L-Aspartic Acid with Ag-Doped ZnO Nanosheets Using Differential Pulse Voltammetry

**DOI:** 10.3390/bios12060379

**Published:** 2022-05-31

**Authors:** Md Mahmud Alam, Abdullah M. Asiri, Mohammad A. Hasnat, Mohammed M. Rahman

**Affiliations:** 1 Center of Excellence for Advanced Materials Research (CEAMR), King Abdulaziz University, P.O. Box 80203, Jeddah 21589, Saudi Arabia; mmalam1@kau.edu.sa (M.M.A.); aasiri2@kau.edu.sa (A.M.A.); 2 Chemistry Department, Faculty of Science, King Abdulaziz University, P.O. Box 80203, Jeddah 21589, Saudi Arabia; 3 Electrochemistry & Catalysis Research Laboratory (ECRL), Department of Chemistry, School of Physical Sciences, Shahjalal University of Science and Technology, Sylhet 3100, Bangladesh; mah-che@sust.edu

**Keywords:** L-aspartic acid, Ag_2_O-doped ZnO nanosheets, voltametric sensor, wet-chemical method, sensitivity, enzyme-free detection

## Abstract

Here, a sensitive voltametric electrochemical sensor probe was fabricated to reliably trace the detection of L-aspartic acid in phosphate-buffered medium using a glassy carbon electrode (GCE) layered with a film of wet-chemically prepared Ag_2_O-doped ZnO nanosheets (NSs). EDS, FESEM, XPS, and X-ray diffraction analyses were implemented as characterizing tools of prepared NSs to confirm the structural and compositional morphology, binding energies of existing atoms, and the crystallinity of synthesized NSs. The differential pulse voltammetry (DPV) was applied to the trace detection of L-aspartic acid, and exhibited a wide detection range of 15.0~105.0 µM, a limit of detection (3.5 ± 0.15 µM), and good sensitivity (0.2689 µA µM^−1^ cm^−2^). Besides these the precious reproducibility, stability, and efficient responses were perceived from the voltametric analysis of aspartic acid. Moreover, the proposed aspartic acid was subjected to experiments to potentially detect aspartic acid in real biological samples. Therefore, the development of an enzyme-free sensor by applying this method will be a smart technical approach in the near future.

## 1. Introduction

Generally, the nonessential amino acids such as isoleucine, methionine, threonine, and lysine biosynthesize in body from aspartic acid. Aspartic acid itself is an amino acid and obtained in the neuroendocrine tissues of vertebrates and invertebrates. It appeared for the first time in the marine mollusks’ nervous system [1,2]. It is known that aspartic acid has a significant role in the development of the nervous system in humans and animals [3,4]. Besides these roles, it also performs in neurotransmission, learning and memory processing in humans [5,6,7]. Therefore, it is an excitatory neurotransmitter and has specific tasks in synapses of the central nervous system (CNS). It also performs various roles in humans such as the production of antibodies, immunoglobulins, healthy DNA, and RNA, regulating the synthesis and release of certain hormones [8]. The deficiency of aspartic acid is rarely perceived because it is biosynthesized by the human body itself. The aspartic acid deficiency is derived from the physical abilities of human, and it results in the decline of the metabolism and other functions in the human body [9,10]. Therefore, several physical syndromes such as energy loss, less attention, irritability, chronic headaches, and food intolerance are promoted by high protein [11,12]. The aspartic acid deficiency may cause injury to the liver, brain, and nervous system in humans [13] in the long run. Therefore, the checking of the aspartic acid levels in humans can save lives, by circumventing these health effects.

The traditional fluorescent method [14,15], infrared spectroscopy [16], spectrophotometric method [17], and chromatography [18] are still in use with some drawbacks such as complicated procedures with heavy and costly equipment, the need for time to prepare samples and analyze them, and them not being portable to in situ detection methods. On the other hand, the electrochemical methods have better advantages when compared to the existing methods [19]. Therefore, the research works are continuously being performed s to develop a reliable and useful electrochemical aspartic acid sensor. Up-to-date, the various research studies have been reported based on the electrochemical methods to aid in the development of an aspartic acid sensor. Recently, an amparometric L-aspartic biosensor has been reported to show a wide detention range (1.0~10.0 mM) with a sensitivity of 0.09 μA mM^−1^ [20], which is appreciable. Unfortunately, this reported L-aspartic acid sensor fails to detect the normal L-aspartic acid levels (35~47 µM) in human blood serum [21]. Another article based on imprinted molecular recognition sites on multi-walled carbon-nanotubes/pencil graphite electrodes has claimed to aid in the electrochemical detection of D- and L-aspartic acid at 0.025 and 0.016 μM as a lower detection limit, respectively, which is an outstanding outcome for the analysis of human serum [22]. Besides this, another D- and L-aspartic acid electrochemical sensor has shown a wider detection range of 3–66 µM that is completely fitted to measure both the deficiency and efficiency of D- and L-aspartic acid levels in human blood samples [23].

According to recent researches, nanomaterials, particularly metal oxides, have attracted researchers for the development of technological tiny electrochemical devices such as an electrochemical sensor. Among the metal oxides, ZnO is a p-type semiconductor with a wide direct optical-band gap of 3.33 eV and exciton binding energy of 60.0 meV at ambient conditions [24]. Therefore, this favorable electrochemical property of ZnO is suited to be used as a sensing electron mediator for the development of sensors. Up-to-date, ZnO has been reported as a sensing substrate to the detection of L-glutamic acid [24], uric acid [25], and thiourea [26] by applying the electrochemical approaches. Meanwhile, Ag_2_O is another p-type semi-conductor consisting of an optical band-gap of 1.46 eV and is found to be used as electrochemical sensors for the electrochemical analysis of thiourea [27], phenol [28], and 3-Methoxyphenol [29] in buffer solution.

In cases of doped materials, Ag-doped ZnO NSs have attracted a great deal of consideration due to their chemical, structural, physical, and optical properties in terms of their large-active surface area, high-stability, high porosity, and permeability, which directly dependent on the structural morphology prepared by reactant precursors for these materials in a basic medium at low-temperatures. In the present research, the NS materials were synthesized by a wet-chemical method using reducing agents at ambient conditions. This technique has several advantages including facile preparation, accurate control of the reactant temperature, being easy to handle, having a one-step reaction, and having high surface area as well as high porosity. Optical, morphological, electrical, and chemical properties of Ag-doped ZnO nanomaterials are of huge significance from the scientific aspect when compared to undoped materials. Non-stoichiometry, mostly oxygen vacancies, promotes the conducting nature of the doped nanomaterials. The formation energy of oxygen vacancies and metal interstitials in semiconductor is very low and thus these defects form eagerly, resulting in the experimentally elevated conductivity of NS materials when compared to other undoped materials. Ag-doped ZnO NSs materials have also attracted considerable interest due to their potential applications in fabricating opto-electronics, electro-analytical methods, the selective detection of assays, sensor devices, hybrid-composites, electron-field emission sources for emission exhibitions, biochemical detections, surface-enhanced Raman properties, etc. Ag-doped ZnO NS material offers an improved performance due to the large-active surface area, which increases conductivity and the current responses during electrochemical investigations. This fabricated electrode is very active and electro-oxidized at the L-aspartic acid with respect to the current against the applied potential when compared to other reported sensors. Due to the oxidation of L-aspartic acid, free electrons and hydrogen ions are generated, which are responsible for enhancing the conductance of the NS-modified working electrode. Due to various benefits, in this study, Ag-doped ZnO NSs prepared with wet-chemical method were used to modify a GCE, which was subjected to analyze L-aspartic acid in a buffer solution. The assembled L-aspartic acid sensor has exhibited appreciable sensitivity, stability, reproducibility, a wide detection range, and an efficient response time in analyzing L-aspartic acid. Beside these advantages, the proposed sensor has shown its reliability in the analysis of real bio-samples.

## 2. Experimental

### 2.1. Materials and Tools

For this study, Sigma-Aldrich (Germany) supplied the precursors of Zn(NO_3_)_2_·6H_2_O and AgCl, which were used to prepare Ag-ZnO NSs, applying the auto-cleave assisted co-precipitation technique in basic medium having pH 0f 10.5. By contrast, L-aspartic acid, disodium phosphate, nomo-sodium phosphate, and adhesive glue (5% Nafion suspension in ethanol) were obtained in analytical grade from Merck, Darmstadt, Germany to conduct the research work. The XPS (Thermo-Scientific, Bremen, Germany, A1-k-α1 radiation sources and 300.0 µm bean size investigated at 200.0 eV) was applied at 10^−8^ Torr pressure on the prepared micro-structures (nano-materials) to analyze the ionization states as well as binding energy of the existing atoms. Later, the structural and elemental compositions of prepared metal oxides were confirmed by using FESEM and EDS analysis. For this purpose, the instrument l JEOL, JSM-7600F (Tokyo, Japan) was used. The crystallinity and particle size of prepared Ag-doped ZnO NSs were evaluated by using a powder X-ray diffractometer. Finally, for the sensor application, the potentiostats/galvanostats (Metrohm Autolab Modules, Herisau, Switzerland) were used as the source of constant potential supply.

### 2.2. Preparation of Ag_2_O-Doped ZnO NSs

For the preparation of doped nanostructure materials, the wet-chemical (co-precipitation) method was used. The advantage of this process is in the preparation of the proper shape and size of doped or un-doped materials at the nano-level. Here, the Ag-doped ZnO NSs were prepared, which were subjected to the co-precipitation technique by using the analytical grade of Zn(NO_3_)_2_·6H_2_O as well as AgCl salts. Initially, for the preparation of this material, a 0.1 M solution of Zn(NO_3_)_2_·6H_2_O and AgCl were prepared separately in two volumetric flasks of 100.0 mL size by using deionized water. On the other side, a 200.0 mL beaker was used to mix this solution taking 50.0 mL each and the resultant solution was stirred rigorously for proper mixing. For the co-precipitation process of these metal ions into the solution, the 0.1 M NH_4_OH was added dropwise in order to increase the pH over 10.5. As we know, over pH 10.5, the dissolved metallic ions would be co-precipitated as metal hydroxides to form the doped nano-crystals of aggregated Ag(OH)·Zn(OH)_2_. Later, the final mixture, which was in a beaker, was kept inside an auto-cleave reactor, where the temperature was fixed at 80 °C for 6 h. The resultant mixture into beaker was placed for 6 h under this condition to complete the co-precipitation in the synthesis of doped nanostructure materials. Then the precipitate was separated from the decanted solution by using filter paper. The precipitate was washed with ethanol, acetone, and water to remove all organic and inorganic parts from the prepared sample as well as unreacted components. The probable chemical reactions are involved in the co-precipitation process, which are presented in the following reactions (Equations (1)–(4)),

NH_4_OH _(s)_ → NH_4_^+^_(aq)_ + OH^−^_(aq)_ (1)

AgCl_(s)_ → Ag^+^_(aq)_ + Cl^−^_(aq)_ (2)

Zn(NO_3_)_2(s)_ → Zn^2+^_(aq)_ + 2NO_3_^−^_(aq)_ (3)

Zn^2+^_(aq)_ + Ag^+^_(aq)_ + 3OH^−^_(aq)_ + H_2_O → Zn(OH)_2_·Ag(OH)·H_2_O ↓ (4)

Here, the prepared aggregated nano-crystals of Zn(OH)_2_·Ag(OH)·H_2_O were dried in an oven at 120 °C for 12 h. The nanocrystals were finally calcined by using the muffle furnace at 500 °C. The prepared metal hydroxides were oxidized to form as metal oxides in the presence of oxygen flow. The following reaction (Reaction (v)) was proposed to form the metal oxide doped nanostructure material during the drying in the muffle furnace. Then the calcined doped nanostructure materials were characterized totally for crystallinity, morphology, size, elemental analysis, band-gap energy etc., by using various conventional methods.

2Ag(OH)·Zn(OH)_2_ + 1/2O_2_ → Ag_2_O·ZnO + 2H_2_O (5)

### 2.3. Fabrication of GCE with NSs

For the fabrication of GCE, the key objective of this study was to modify the electrode surface with prepared Ag-ZnO NSs and then apply it for the development of an efficient working electrode of the proposed L-aspartic acid electrochemical sensor probe by the electrochemical approach. So, the tiny surface area of GCE (0.0314 cm^2^) was fabricated with slurry of Ag-doped ZnO NSs, which was prepared into the ethanol solution. The geometric surface area of an electrode was calculated from the equation (A = πr^2^), where r is equal to 0.1 cm, and r is the radius of the GCE. Then the modified electrode was kept under ambient conditions with air drying for 10 min. For the improvement of stability of the fabricated layer onto GCE, a conducting coating binder as 5% Nafion was used. The net type binder was used as a chemical glue to stick the materials onto GCE for electrochemical analysis. This fabricated electrode with NSs and Nafion was kept under air drying for 30 min to stabilize the thin surface of the materials onto the electrode. Finally, the electrode was prepared with the Ag-doped ZnO NSs as an Ag-doped ZnO NSs/Nafion/GCE sensor probe to practically use in the electrochemical investigation. Here, the Pt-wire and Ag/AgCl (sat. KCl) were used as a counter and reference electrode, respectively, in this analysis. Then, the analyte solution (L-aspartic acid) was used to prepare a series of solutions ranging from 15 to 105 µM and was applied to the electrochemical characterization of the assembled sensor. Mono- and disodium phosphate solution (0.1 M) were used for dilution into deionized water for the formation of the phosphate-buffered solutions with different pH values ranging from 5.7 to 8.0. Finally, 10.0 mL of these prepared phosphate-buffered solutions were used to carry out the relevant investigations.

## 3. Results and Discussion

### 3.1. Structural Morphology of Ag_2_O-Doped ZnO NSs

**Figure 1:**
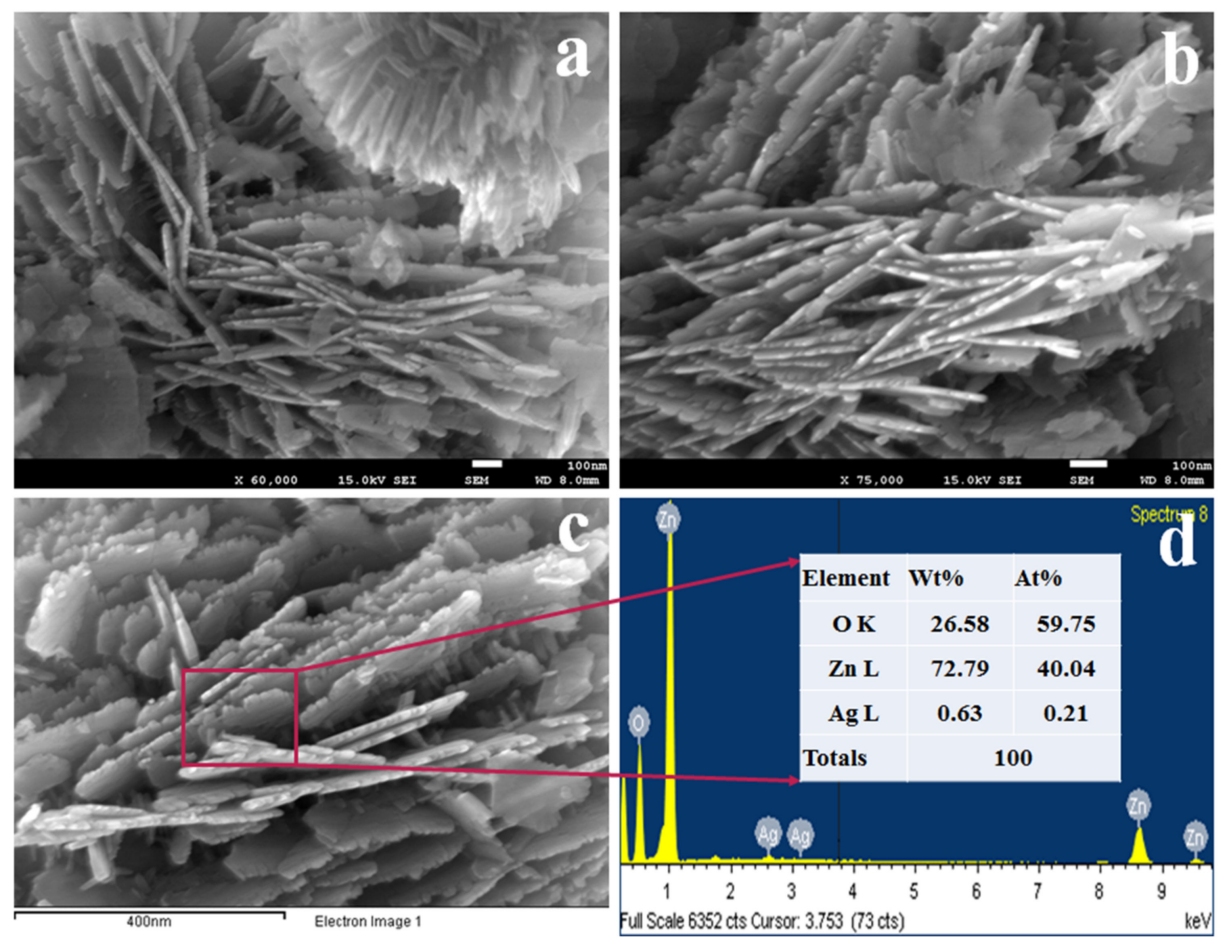
Morphological and elemental analyses of nanomaterial. (**a**,**b**) the low and high magnifying images of synthesized Ag/ZnO nanomaterial, (**c**) image of EDS, and (**d**) the elemental compositions of prepared NSs.

The wet-chemically prepared Ag/ZnO nanomaterials was subjected to image capture, with magnified images on FESEM attached with EDS as perceived in Figure 1a,b.

From the Figure 1, it is seen that Ag and ZnO metal oxides are regularly aggregated to each other to form sheet-like structures, and these are called nanosheets. The EDS image of synthesized nano-materials as seen in Figure 1c is also confirmed to have the nano-sheet structure. Therefore, the nano-materials can be considered as nanosheets (NSs). The elemental compositions evaluated by EDS perceived in Figure 1d are 26.58% O, 72.79% Zn, and 0.63% Ag as weight. It also evidenced these materials as Ag-doped ZnO NSs.

### 3.2. X-ray Diffraction of Ag-Doped ZnO NSs

**Figure 2:**
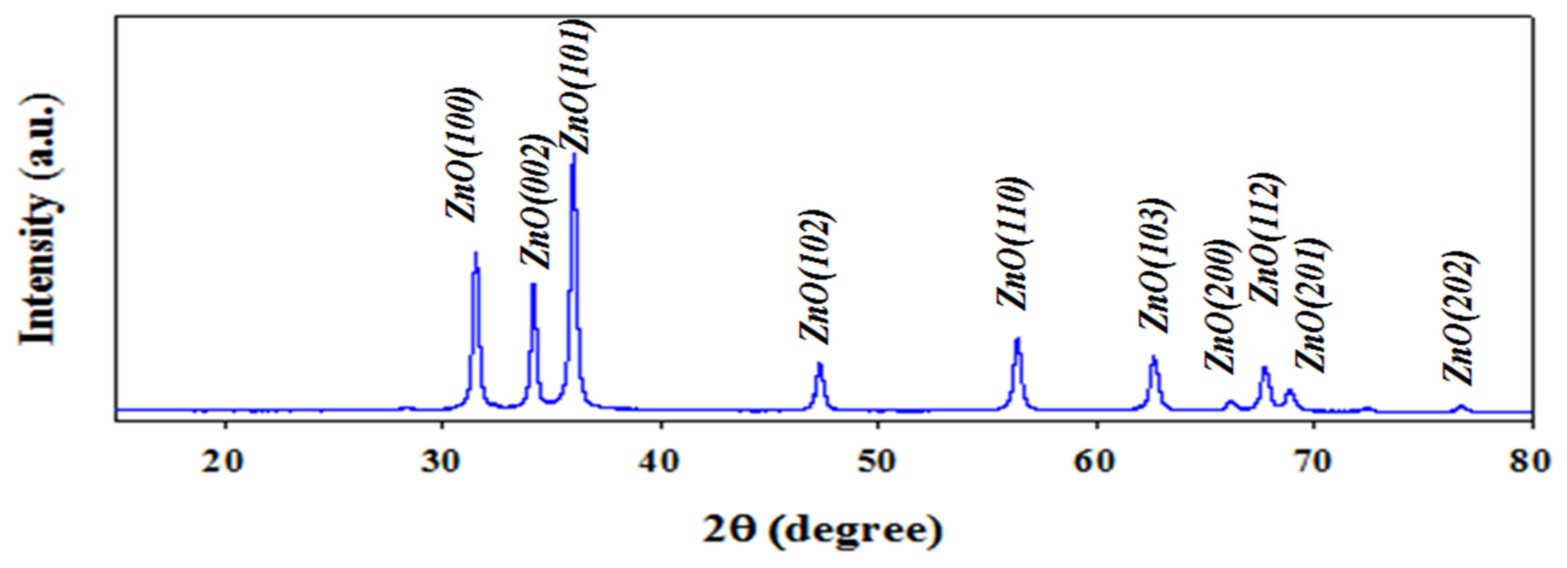
The XRD analysis of Ag-doped ZnO NSs at 2θ degree in the range of 15–80°.

The X-ray diffraction of Ag-doped ZnO NSs was analyzed at 2θ = 15~80° as illustrated in Figure 2 and it is confirmed the existence of crystalline plans of ZnO. As perceived, the X-ray diffraction pattern shows crystalline plans of ZnO of (100), (002), (101), (102), (110), (103), (200), (112), (201), and (202) [30,31]. The majority of components (ZnO) in the synthesized Ag-doped ZnO NSs are clarified by JCPDs No. 0036-1451.

The crystallographic dimension of the synthesized Ag-doped ZnO NSs can be evaluated by applying Scherrer Equation as in below.

D = (0.94 λ)/(β Cosθ)(6)

Herein λ (wavelength of X-ray radiation) and β (the width at half of peak). The dimension of NSs at ZnO (100) is 36.01 nm.

### 3.3. XPS Analysis of Ag-Doped ZnO NSs

**Figure 3:**
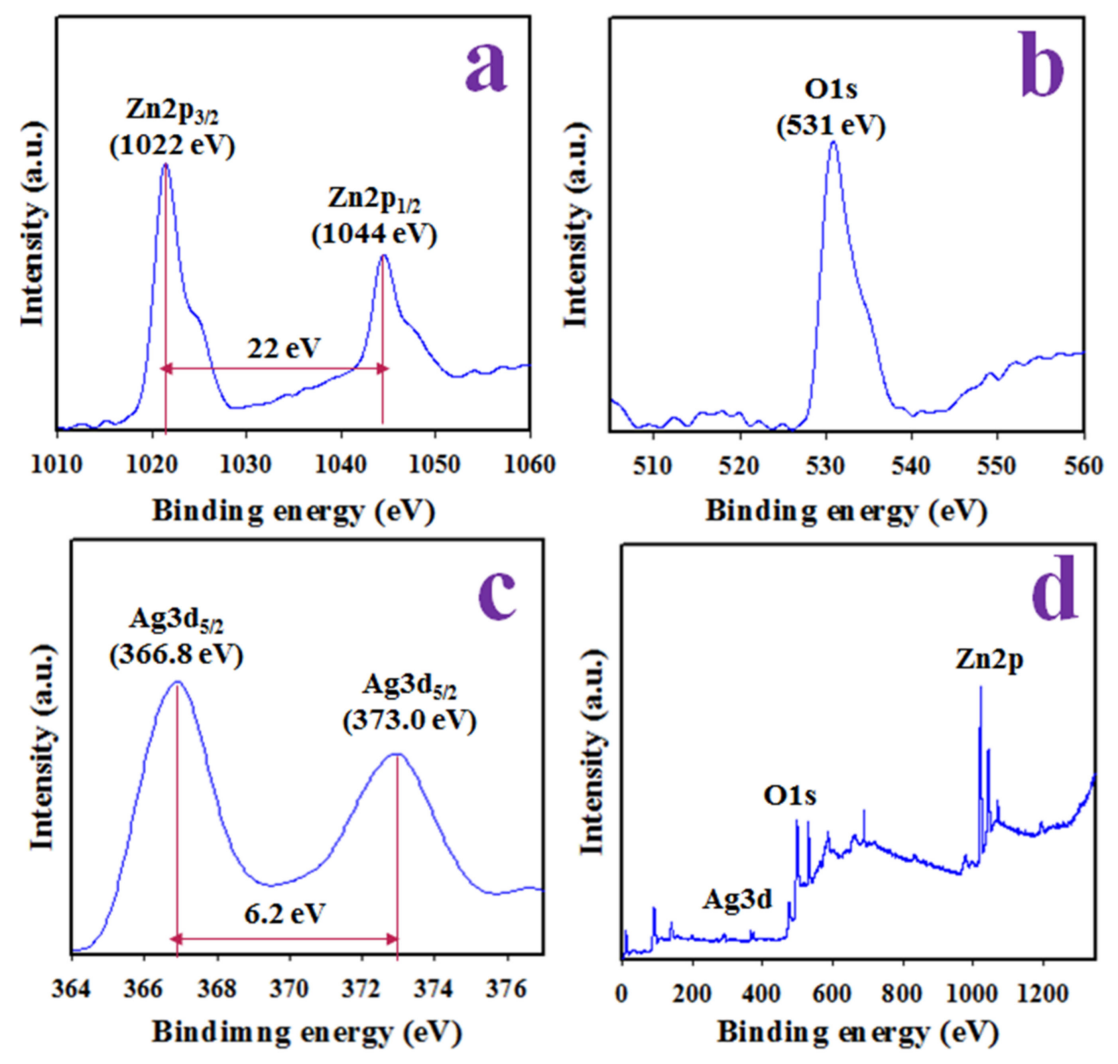
XPS analysis. (**a**) The spin orbitals of Zn2p level, (**b**) O1s orbit, (**c**) Ag3d_5/2_ and Ag3d_5/2_ spin orbitals, and (**d**) surveyed XPS spectrum of Ag-doped ZnO NSs.

To identify the elements, their oxidation states, and the contamination of Ag-doped ZnO NSs, X-ray photoelectron spectroscopy (XPS) is the better option. Therefore, the synthesized NSs were subjected to XPS investigation, and the resultant wide-scan survey spectrum is illustrated in Figure 3d.

The Zn2p level orbital is sub-divided into two spin orbitals which exhibited peaks at 1022 and 1044 eV, corresponding to Zn2p_3/2_ and Zn2p_1/2_ with a separation of 22 eV, which is evidence of the presence of Zn^2+^, as explored in Figure 3a [32]. The peak at 531 eV illustrated in Figure 3b can be assigned as the lattice oxygen of synthesized Ag-doped ZnO NSs and presents O^2−^ ionization [33]. Besides, Ag3d XPS is doubled at 366.8 and 373.0 eV with respect to Ag3d_5/2_ and Ag3d_3/2_, with a separation of 6.2 eV, as demonstrated in Figure 3c, provides the evidence of existence Ag^+^ species [34].

### 3.4. Electrochemical Characterization of Working Electrode

**Figure 4:**
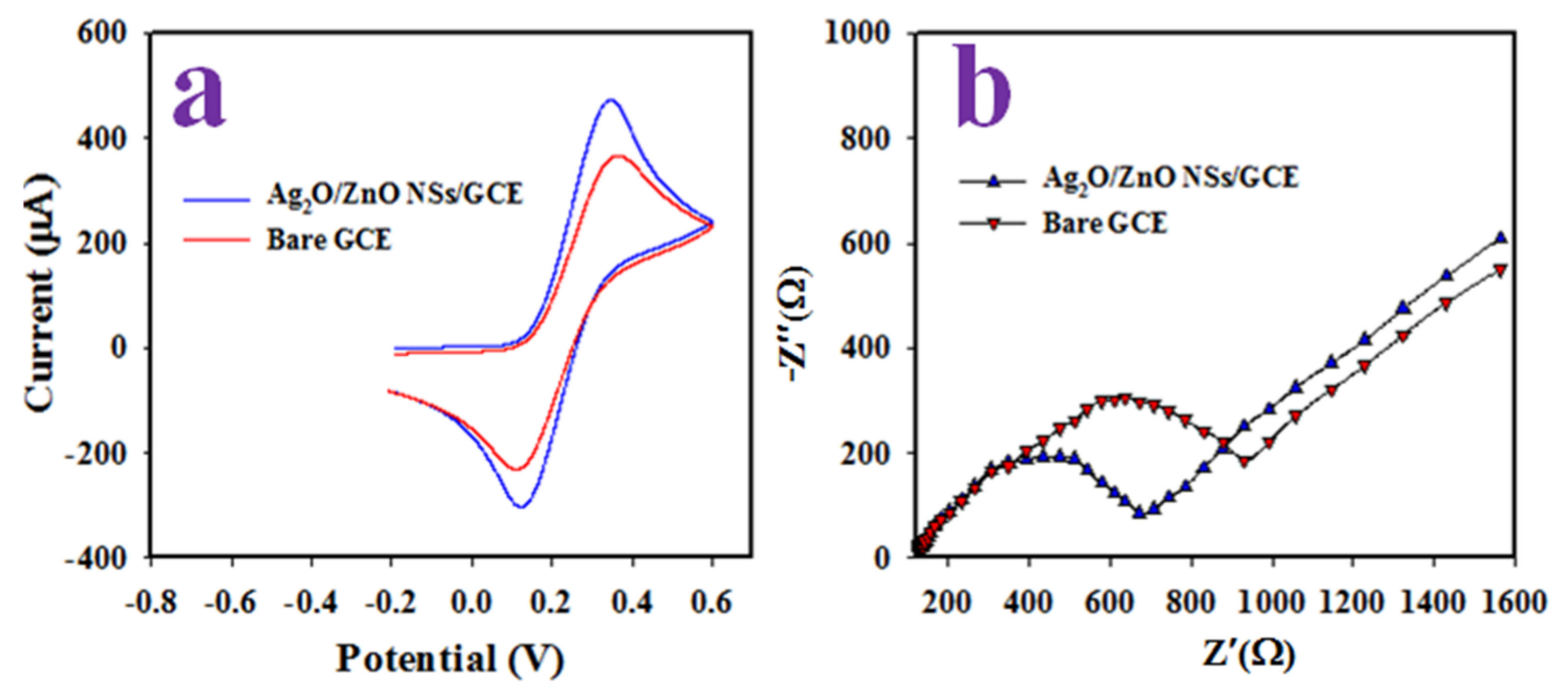
(**a**) The cyclic voltammetry of bare and Ag_2_O-doped ZnO NSs modified GCE in 0.1 mM [Fe(CN)6^3−^/[Fe(CN)6^4−^] in 7.0 pH buffer and (**b**) the electrochemical impedance spectroscopy (EIS) of bare and Ag_2_O-doped ZnO NSs modified GCE in 0.1 mM [Fe(CN)_6_^3−^/[Fe(CN)_6_^4−^] in 7.0 pH buffer.

The electrochemical properties of the Ag-doped ZnO NSs/GCE working electrode was estimated by applying cyclic voltametric (CV) analysis of 0.1 mM [Fe(CN)6^3−^/[Fe(CN)6^4−^] redox couples in a 30 mL phosphate-buffered solution of 7.0 pH at ambient conditions as presented in Figure 4a. The oxidation and reduction peaks observed with the modified were observed at +0.32 and +0.12 V (shown in Figure 4a), respectively. On the other hand, the bare GCE exhibited comparatively lower oxidation/reduction peaks, with the current positioned at +0.10 and 0.36 V, respectively, with a wider peak separation of +0.26 V when compared to coated GCE (+0.20 V). It is predicted that the high electron mobility for Ag-doped ZnO NSs/GCE will assist in an electrochemical analysis of an analyte due to its high oxidation and reduction peak currents with a narrow peak-separation (∆Ep). This observation can confirm the enhanced electron transfer rates while subjected to carry on an electrochemical reaction [35]. However, the measurement of conductance of the Ag-doped ZnO NSs/GCE might be more appropriate property to assess the oxidation/reduction capability of an analyte. Therefore, the electrochemical impedance spectroscopy (EIS) using 0.1 mM [Fe(CN)6^3−^/[Fe(CN)6^4−^] in 30 mL 7.0 pH buffer solution was estimated with respect to Ag-doped ZnO NSs/GCE, which is illustrated in Figure 4b. It can be seen that the semi-circle diameter is smaller for Ag-doped ZnO NSs/GCE (729 Ω) to bare GCE (968 Ω), which provides the evidence for a lesser charge-transfer resistance (Rct) for the working electrode. Thus, it can be predicted that the modified Ag-doped ZnO NSs/GCE working electrode is capable of enhancing the charge-transfer for the electrochemical detection of an analyte. This concept has reported previously [36].

**Figure 5:**
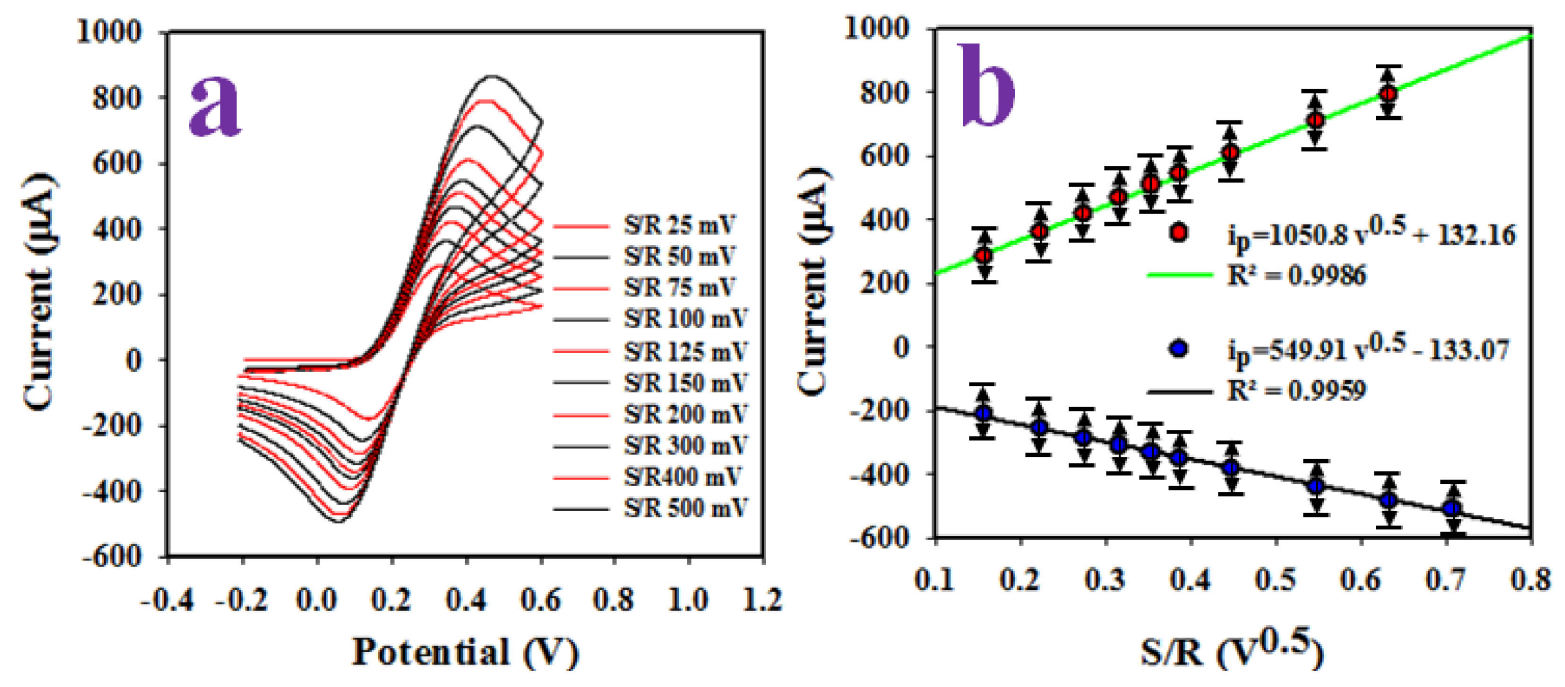
(**a**) Scan rate variant CVs recorded with assembled Ag_2_O-doped ZnO NSs electrode for 0.1 mM [Fe(CN)6^3−^/[Fe(CN)6^4−^], in 7.0 pH buffer, (**b**) V^0.5^ versus i_p_ (oxidation/reduction).

Figure 5a illustrates the scan rate (SR) variant CVs recorded with GCE modified Ag-doped ZnO NSs for 0.1 mM [Fe(CN)6^3−^/[Fe(CN)6^4−^] in a 30 mL buffer solution of pH 7.0. As shown in Figure 5a, the oxidation peaks at +0.32 V and reduction peaks at +0.12 V increased and decreased linearly, respectively, as the SR is applied in a range of 25~500 mV/s. The oxidation peak current (i_pa_) and reduction peak current (i_pc_) were plotted against square root of SR as demonstrated in Figure 5b. The relationship can be expressed as i_p_ = 1050.8 V^0.5^ + 132.16; R^2^ = 0.9986 for oxidation and ip = 549.9 V^0.5^−133.1; R^2^ = 9959 for the reduction processes, respectively. The plots demonstrated good linearity in both cases suggesting that the oxidation and reduction of 0.1 m [Fe(CN)6^3−^/[Fe(CN)6^4−^] with an assembled working electrode were diffusion-controlled processes.

### 3.5. Voltammetric Detection of L-Aspartic Acid

**Figure 6:**
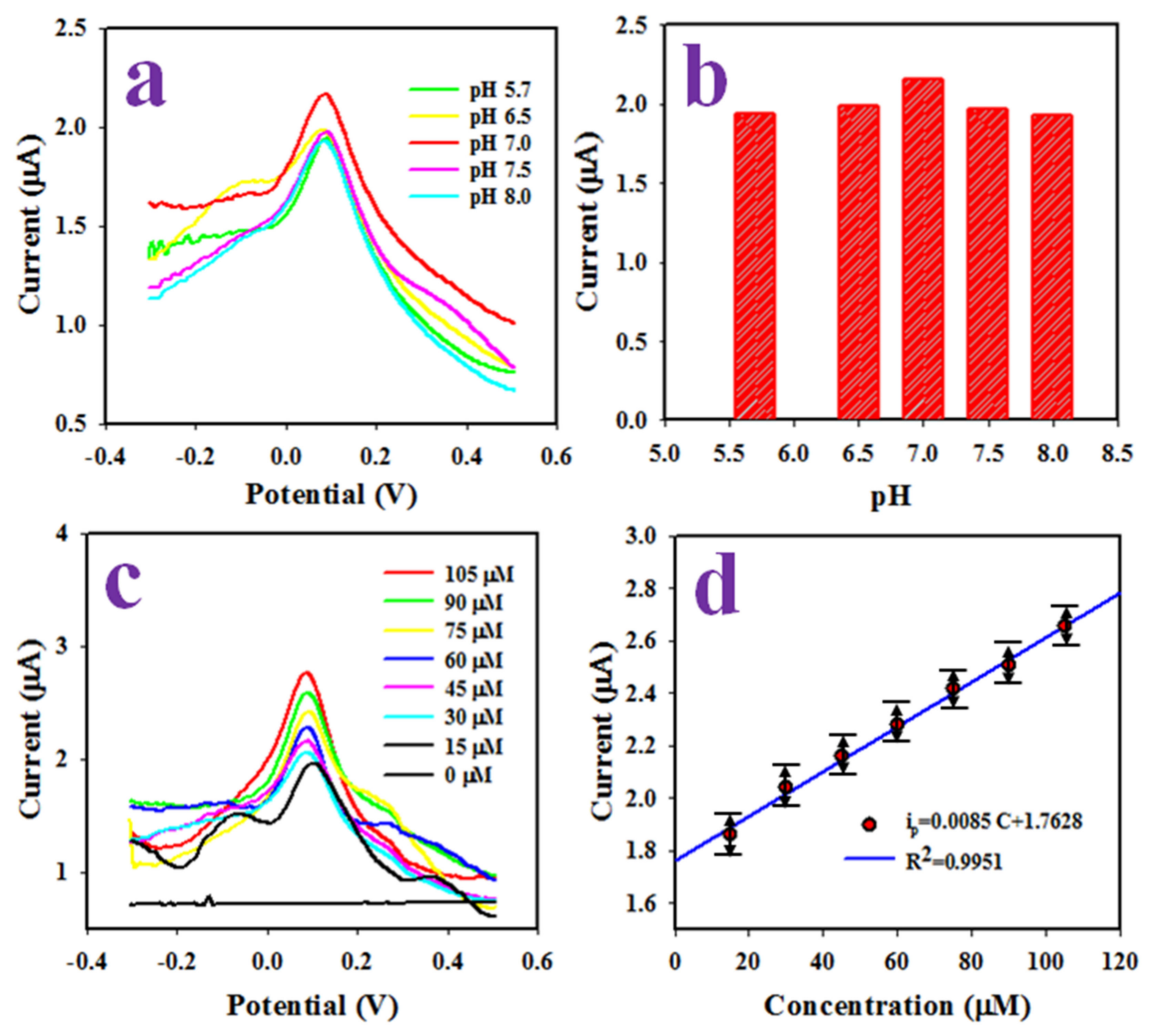
(**a**) The pH effects on the DPV analysis of L-aspartic acid, (**b**) the bar diagram of pH effect in electrochemical analysis of L-aspartic acid, (**c**) the DPV analysis of L-aspartic acid in pH 7.0 based on the concentration variation from low to high, and (**d**) the calibration of L-aspartic acid sensor.

The differential pulse voltametric (DPV) method has applied to the analysis of L-aspartic acid. The assembled working electrode based on Ag-doped ZnO NSs/GCE used to detect L-aspartic acid at 45.0 µM concentration and applied a potential range of −0.3 to +0.5 V in a buffer solution of various pH values, as illustrated in Figure 6a. Figure 6a presents the optimization of the pH (5.7~8.0) value of the buffer solution, which has the highest oxidation peak current at 45.0 µM L-aspartic acid, and it demonstrates the highest oxidation peak current, observed at pH 7.0 in an analysis of L-aspartic acid, applying DPV as illustrated the bar diagram at Figure 6b. In the next experiment, a range (15.0~105.0 µM) of L-aspartic acid solution in a buffer solution of pH 7.0 was subjected to the electrochemical analysis as presented in Figure 6c where, the oxidation peak current being found to be increased when going from a low to high concentration of L-aspartic acid.

As it is observed that the current density is enhanced with increasing concentrations of the analyte (L-aspartic acid), the electrochemical oxidation of the analyte occured. To calibrate this L-aspartic acid sensor, the obtained currents at the peak position in L-aspartic acid electrochemical analysis were plotted against the concentration of L-aspartic acid, which is explored in Figure 6d. From the obtained current data, the i_p_ (µA) = 0.0085 C (µM) + 1.7628 (R^2^ = 0.9951) equation can be formulated, which is linear at a concentration range of 15.0–105.0 µM, which is defined as the dynamic range (LDR) for L-aspartic acid detection. Obviously, the resultant LDR is a wider range of concentration. The sensitivity of the sensor is an important analytical parameter and measured using the LDR slope (0.0085 µA µM^−1^) and GCE surface area (0.0314 cm^2^). Thus, the resulting sensitivity (0.2689 µA µM^−1^ cm^−2^) is good and appreciable. The limit of detection (LOD) of L-aspartic acid is calculated using a signal/noise ratio of three. The obtained LOD is calculated as 3.5 ± 0.15 µM; this result might be satisfactory.

**Figure 7:**
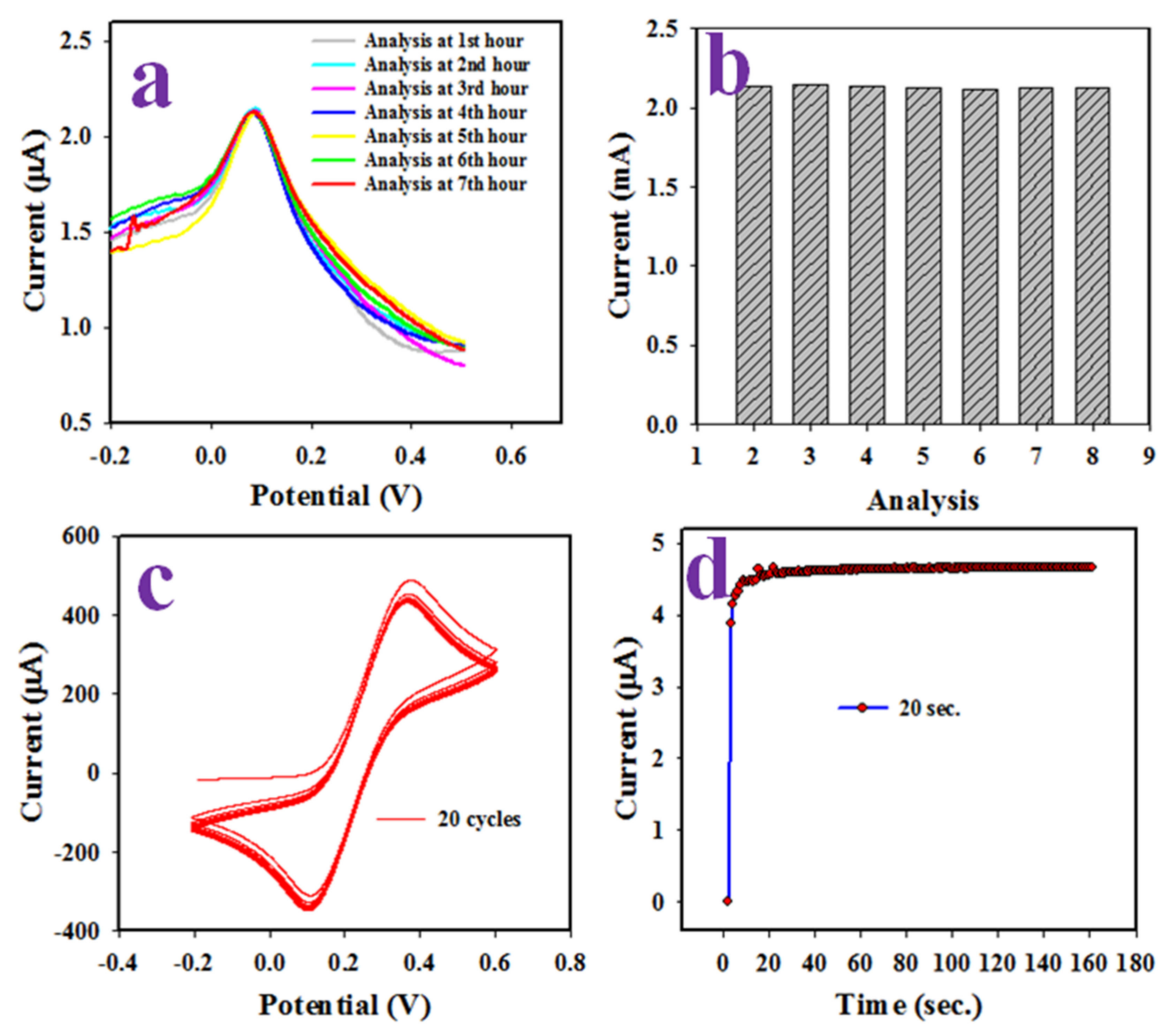
Optimization of sensor probe of Ag_2_O doped ZnO NSs/GCE. (**a**) Reproducibility of L-aspartic acid sensor, (**b**) reproducibility in bar diagram, (**c**) the stability of L-aspartic acid sensor and (**d**) response time.

The stability between the layers of NSs and GCE in the DPV electrochemical analysis of L-aspartic acid is a requirement to develop a reliable electrochemical sensor. Therefore, the reproducibility of the L-aspartic acid sensor using Ag-doped ZnO NSs/GCE was tested in 45.0 µM L-aspartic acid in the buffer solution of pH 7.0 illustrated in Figure 7a and the bar diagram at Figure 7b. The obtained results were found to be undistinguishable under the similar conditions of the experiments. It appears from Figure 7a,b that the electrochemical sensor Ag-doped ZnO NSs/GCE can analyze L-aspartic acid reliably. The stability of this L-aspartic acid sensor is another essential criteria (parameter). It was evaluated by investigating 0.1 mM [Fe(CN)6^3−^/[Fe(CN)6^4−^] in 7.0 pH of buffer phase as it could be perceived from Figure 7c. Thus, L-aspartic acid sensor was found to be quite stable while performing at 20 cycles in the voltametric electrochemical analysis of 0.1 mM [Fe(CN)6^3−^/[Fe(CN)6^4−^]. Therefore, it can be predicted that Ag-doped ZnO NSs/GCE sensor will able to work for a long duration continuously. The response time, which is an efficiency-measuring parameter of an electrochemical sensor, is defined as the period needed to complete the analysis of an analyte. Thus, the L-aspartic acid sensor response time was investigated in the buffer solution of pH 7.0 at 45 µM of L-aspartic, by applying a three-electrode system electrochemical method, as shown in Figure 7d, and an appreciable time (20 s) to complete the electrochemical analysis of L-aspartic acid was found, which provides the high efficiency of the L-aspartic acid sensor.

**Scheme 1:**
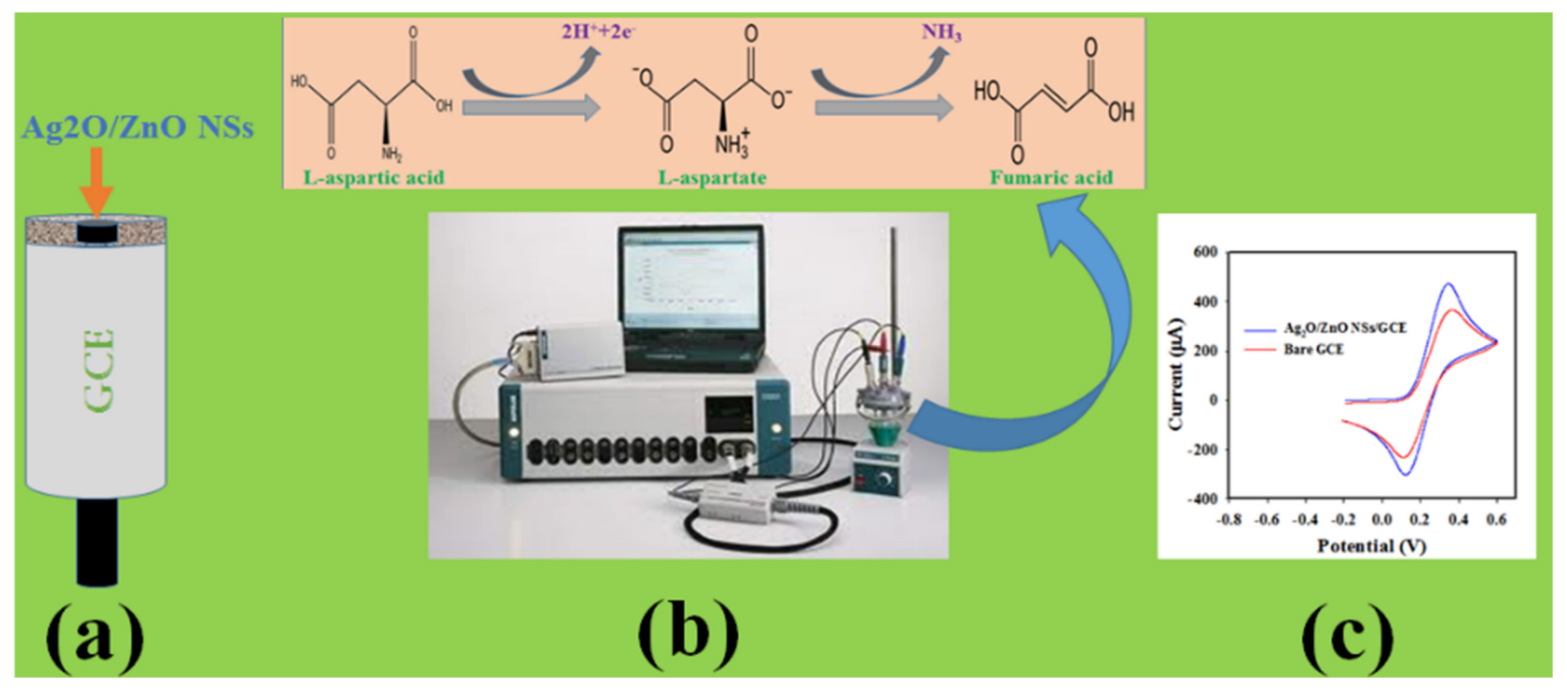
Schematic representation of detection mechanism. (**a**) Modification of GCE with Ag-doped ZnO NSs, (**b**) sensor assembly for cyclic voltametric analysis of L-aspartic acid to fumaric acid, (**c**) Analyzed data.

However, the proposed electrochemical oxidation of L-aspartic acid to fumaric acid using Ag_-_doped ZnO NSs/GCE as a working electrode is perceived in Scheme 1. As illustrated in Scheme 1, the L-aspartic acid molecules are oxidized to L-aspartate on the surface of modified GCE at an applied potential measured through an Auto-lab instrument. During the further oxidation of L-aspartate, fumaric acid is produced as reported previously [37]. Due to the oxidation of L-aspartic acid, free electrons and hydrogen ions are generated, and are responsible for enhancing the conductance of the working electrode, and electrochemical responses can be recorded.

For the reliability of this investigation, similar reported studies have been compared with our present work as illustrated in Table 1 [37,38,39], considering the sensor analytical parameters such as sensitivity, LOD, and LDR. As perceived in Table 1, our studied L-aspartic acid sensor based on Ag-doped ZnO NSs/GCE is shown to have appreciable performances and a confirmed good reliability. This approach is introduced a new route to the development of the sensor probes with various nanostructure or nanoscale materials [40,41,42,43,44,45,46,47,48] for the trace detection of unsafe chemicals by using electrochemical method for the safety of healthcare and environmental fields in a broad scale.

### 3.6. Analysis of Real Samples

Finally, the assembled L-aspartic acid sensor was used to analyze the collected bio-samples such as human, rabbit, and mouse serum, using the DPV method. The obtained results are illustrated in the Table 2, which seem to be quite satisfactory and acceptable.

## 4. Conclusions

Finally, the fabricated sensor based on Ag_-_doped ZnO NSs/GCE was successful implemented to detect L-aspartic acid in phosphate-buffered solution at pH 7.0. It is exhibited good sensitivity (0.2689 µA µM^−1^ cm^−2^), a wider detection range (15.0~105.0 µM), with the lowest detection limit (3.5 ± 0.15 µM), which are very good parameters in analyzing human serum practically. Besides, it was shown to aid significantly in the analysis of the biological samples with a high reproducibility, stability, and a short response time. This potential electrochemical approach would bring a turning point for the development of portable electrochemical sensor probes for real-time monitoring of biological analysis in broad scales for early diagnosis in the biomedical and healthcare fields.

## Figures and Tables

**Table 1:** The comparison of the analytical performances of L-aspartic acid sensors based on Ag-doped ZnO NSs/GCE.

Modified Electrode	Method	DOL *	LDR ^#^	Sensitivity	Ref.
Fe_3_O_4_@ZnO NPs/GCE	I–V	97.0 pM	0.1 nM~10 μM	0.1266 µA µM^−1^ cm^−2^	[37]
Au NPs–PT	fluoresce	32.0 pM	75.0 pM~6.0 µM	---	[38]
MWCNT/PGOE	DPV	16.0 pM	0.15–8.9 µM	---	[39]
Ag_2_O-ZnO NSs/GCE		3.5 µM	15.0~105.0 µM	0.2689 µA µM^−1^ cm^−2^	This work

* DL (detection limit), ^#^ LDR (linear dynamic range), µM (micromole), mM (millimole).

**Table 2:** Analyses of real biological samples with an Ag_2_O-doped ZnO NSs/GCE sensor probe by the electrochemical method.

Sample	Added L-Aspartic Conc. (µM)	Measured L-Aspartic Conc. by Ag_2_O Doped ZnO NSs/GCE (µM) ^a^			Average Recovery ^b^ (%)	RSD ^c^ (%) (*n* = 3)
		R1	R2	R3		
Human serum	45	44.23	44.15	43.96	98.03	0.60
Mouse serum	45	43.76	43.89	44.17	97.64	1.20
Rabbit serum	45	42.93	43.27	44.07	96.49	1.02

^a^ Means of three repeated determination (signal to noise ratio of 3) with Ag_2_O-doped ZnO NSs/GCE. ^b^ Concentration of L-aspartic acid determined/concentration taken. (Unit: µM). ^c^ Relative standard deviation value indicates precision among three repeated measurements (R1, R2, R3).

## Data Availability

Data is contained within the article.
